# Supplementary material for: Aggregation-induced emission luminogens for image-guided surgery in non-human primates
Source: Nat Commun. 2021 Nov 10;12:6485. doi: 10.1038/s41467-021-26417-2 (PMC9632329; doi:10.1038/s41467-021-26417-2)
Supplement: Supplementary file 1 — Supplementary Info [file 41467_2021_26417_MOESM1_ESM.pdf]

# Supplementary information

## Aggregation-Induced Emission Luminogens for Image-Guided Surgery in Non-Human Primates

Danni Zhong[1,2‡], Weiyu Chen[3,8‡], Zhiming Xia[2, 4,‡], Rong Hu[5,‡],Yuchen Qi[2,‡], Bo Zhou[2], Wanlin Li[2], Jian He[2], Zhiming Wang[5], Zujin Zhao[5], Dan Ding[6], Mei Tian[11], Ben Zhong Tang[5,7*], and Min Zhou[1,2,9,10*],

[1]Eye Center, the Second Affiliated Hospital, Zhejiang University School of Medicine, Hangzhou 310009, China.

[2]Institute of Translational Medicine, Zhejiang University, Hangzhou, 310009, China.

[3]The Fourth Affiliated Hospital, Zhejiang University School of Medicine, Yiwu, 320000, China.

[4]Department of Nuclear Medicine, the Second Affiliated Hospital, Zhejiang University School of Medicine, Hangzhou 310009, China.

[5]NSFC Center for Luminescence from Molecular Aggregates, SCUT-HKUST Joint Research Institute, State Key Laboratory of Luminescent Materials and Devices, South China University of Technology, Guangzhou, 510640 China

[6]Key Laboratory of Bioactive Materials, Ministry of Education, and College of Life Sciences, Nankai University, Tianjin 300071, China

[7]Department of Chemistry, Hong Kong Branch of Chinese National Engineering Research Center for Tissue Restoration and Reconstruction, State Key Laboratory of Neuroscienceand Division of Biomedical Engineering, The Hong Kong University of Science and Technology (HKUST), Clear Water Bay, Kowloon, Hong Kong, China

[8]Molecular Imaging Program at Stanford, Department of Radiology, Stanford University, Stanford, CA 94305-5427, USA

[9]Cancer Center, Zhejiang University, Hangzhou 310009, China.

[10]State Key Laboratory of Modern Optical Instrumentations, Zhejiang University, Hangzhou, 310058, China

[11]Department of Nuclear Medicine, Shandong Provincial Hospital Affiliated to Shandong First Medical University, Jinan, 250021, China.

‡These authors contributed equally to this work.

*Corresponding authors. Email: zhoum@zju.edu.cn; tangbenz@ust.hk

## Content of Experiments, Tables and Supplementary Figures


Table. 1. Post-inspection of the rhesus macaque.

Table. 2. Changes in the bodyweight of rhesus macaque 1-day before and 180-day after operations.

Table. 3. Systematical Comparison of ICG dye and AIEgens.

Table. 4. Advanced performances and limitations of AIEgens and typical contrast agents.

Fig. S1. Characterizations of folic-AIEgen.

Fig. S2. Cell viabilities of different cells (HEK293, HL7702, 4T1, and fibroblast cells) after incubation with series concentrations of folic-AIEgen for 24 h, respectively.

Fig. S3. Folic-AIEgen for targeting cell imaging.

Fig. S4. Histological images of the major organs from the mice at 30 days after surgical operation.

Fig. S5. Histological images of the major organs from the rabbits at 30 days after surgical operation.

Fig. S6. Histological images of the major organs from the mice at 30 days after different treatments.

Fig. S7. Blood test results (a-l) for the rhesus macaques before and after the surgical operation.

Fig. S8. Specific tumor targeting ability of folic-AIEgen in the intraperitoneal SKOV3 xenograft mouse model.

Fig. S9. Histological images of the major organs from the tumor-bearing mice at 30 days after surgical operation.

Fig. S10. Blood test results for the SKOV3 tumor-bearing mice after the intravenous injection.

Fig. S11. Targeted imaging of folic-AIEgen in metastatic liver tumor.

Fig. S12. White light and bioluminescence images of intraperitoneal SKOV3 and Hela tumors bearing mice after surgery.

Fig. S13. Survival rate of intraperitoneal SKOV3 and Hela tumors bearing mice with or without surgery.

Fig. S14. Comparison of fluorescence characteristics and stability between ICG and folic-AIEgen.

Fig. S15. Schematic illustration of ICG and folic-AIEgen guided SLN dissection in nude mice.

Fig. S16. Biodistribution of ICG and folic-AIEgen in nude mice after intravenous administration.

| Behaviors check list | Results |
|---|---|
| Eating | Normal |
| Drinking | Normal |
| Urination | Normal |
| Defecation | Normal |
| Sleeping | Normal |
| Activity | Normal |
| Grooming | Normal |
| Neurological | Normal |

**Supplementary Table 1. Post-inspection of the rhesus macaque.** No abnormalities were observed in behaviors of rhesus macaque after operations, including eating, drinking, urination, defecation, sleeping, activity, grooming, and neurological status.

| Date | Body weight （Kg） |
|---|---|
| Before Surgery | 5.80 |
| Post-Surgery: Day 1 | 5.88 |
| Post-Surgery: Day 7 | 5.98 |
| Post-Surgery: Day 30 | 6.22 |
| Post-Surgery: Day 90 | 6.38 |
| Post-Surgery: Day 180 | 6.55 |

**Supplementary Table 2. Changes in the bodyweight of rhesus macaque 1-day before and 180-day after operations.** Bodyweight of the monkey was monitored

1-day before and 180-day after the surgical operation. Over the whole period, no greater fluctuations were observed after 180-day treatment (6.55 kg) than before treatment (5.80 kg).

| Properties | ICG, NIR fluorescence ~850 nm | AIEgens, visible fluorescence ~540 nm |
|---|---|---|
| Component | Small organic dyes (<1 KD) | Fluorescent nanoparticls (Diameter: ca 20 nm) |
| Dispersibility | Dispersed in sterile water but easily aggregated in physiological saline | Well dispersed in both sterile water and physiological saline |
| Fluorescent stability | 1. Decrease to <40% of initial fluorescence intensity within 24 h at 37 ℃; 2. Rapid photobleaching to ~52% in 105 min under continuous laser irradiation | 1. Remain stable during 3-day storage; 2. Possess superior photostability, preserving ~80% during 105 min continuous laser irradiation |
| Real-time surgical operation | 1. NIR fluorescence imaging system, including NIR excitation light, collection optics, filtration, NIR camera, color camera; 2. Assisted by surgeon's guidance due to undetectable NIR fluorescence seen by naked eyes | Portable UV lamp |
| Medical equipment cost | High cost (~100,000 US dollars) | Low cost (100-500 US dollars) |
| Medical training | Long-term training time | No additional training Simple operation procedure |

**Supplementary Table 3. Systematical Comparison of ICG dye and AIEgens.**

| Typical example | Method | Diameter | Advanced performances | Limitations |
|---|---|---|---|---|
| $^{99m}$Technetium nanocollids | Nuclear imaging | 50~3000 nm | Detect deep-seated targets (~ 25 mm) | 1. Large sizes; 2. Slow clearance from injection site; 3. Slow physiologic transport within lymphatics |
| ICG | MR fluorescence imaging | <1 KD | Detect superficially located targets (~5 mm) | Extravasation and nonspecific tissue scattering |
| Evans blue | Visible blue color | <1 KD | Aid visual identification under white-light illuminations | 1. Limited tissue penetration; 2. Lose visibility when intraoperative bleeding; 3. Extravasation and nonspecific tissue scattering |
| AIEgens | Naked eye visible fluorescence | ~20 nm | 1. Aid visual identification under UV-light illuminations; 2. Visualize the draining lymphatics in real time | Limited tissue penetration |

**Supplementary Table 4. Advanced performances and limitations of AIEgens and typical contrast agents.**

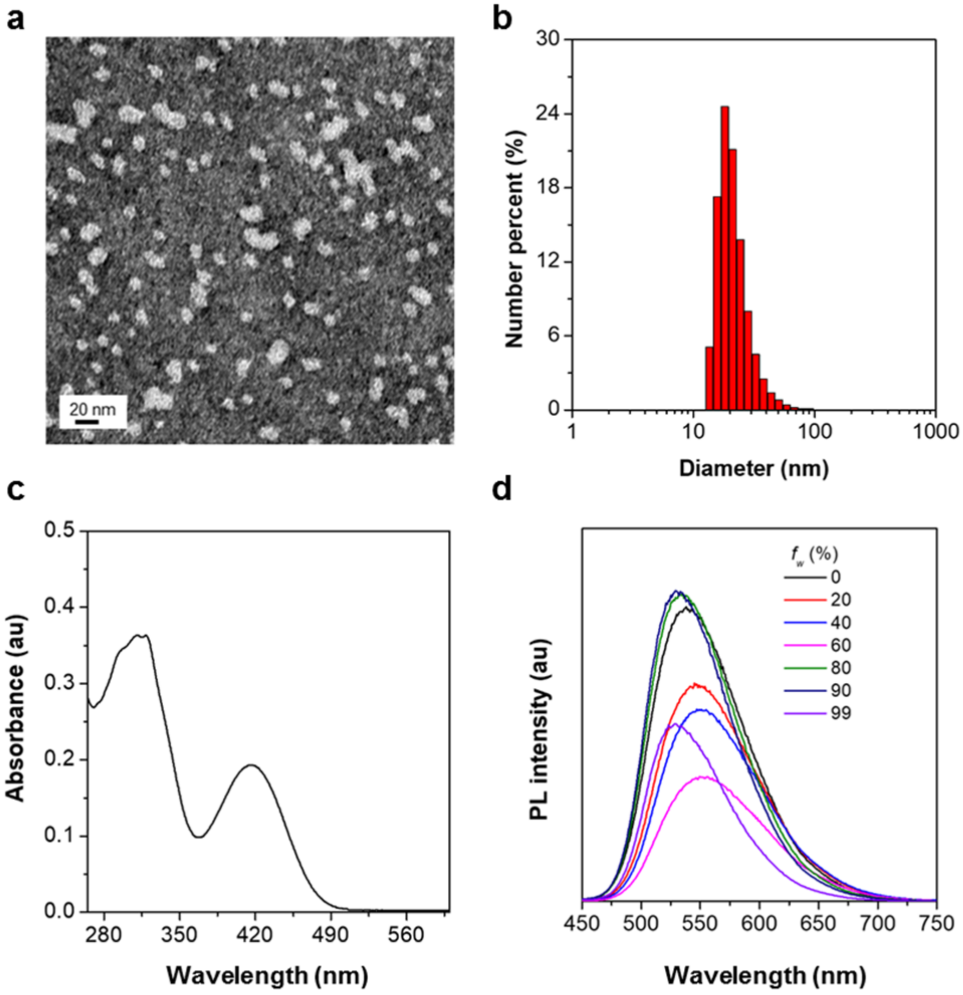


**Supplementary Figure 1. Characterizations of folic-AIEgen.** (a) Transmission electron microscopy (TEM) image. The experiment was repeated at least three times. (b) DLS, and (c) UV-vis spectra of the as-prepared folic-AIEgen. (d) Photoluminescence of AIEgen in tetrahydrofuran with different fraction of water.

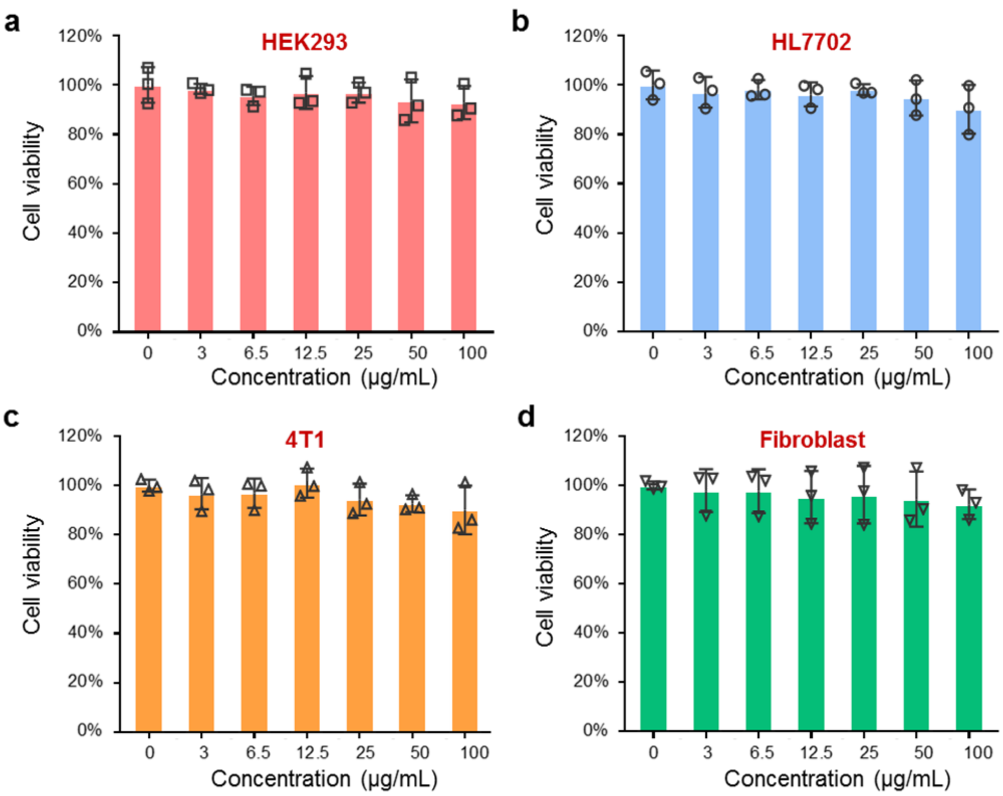


**Supplementary Figure 2. Cell viabilities of different cells (HEK293, HL7702, 4T1, and fibroblast cells) after incubation with series concentrations of folic-AIEgen for 24 hours, respectively.** No significance was observed in terms of cell viability among four types of cells incubated with different concentrations of folic-AIEgen. Data are means ± SD, n = 3.

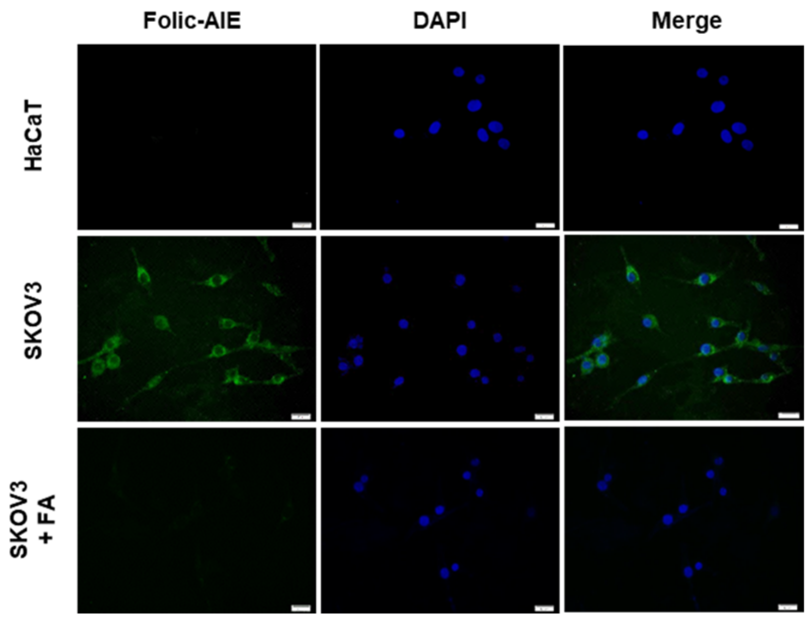


**Supplementary Figure 3. Folic-AIEgen for targeting cell imaging.** SKOV3 cells with over-expressing FR displayed a significant enrichment of folic-AIEgen after incubation (middle) and free folic acid competitively inhibited the uptake of folic-AIEgen (bottom). HacaT cells (with a middle expression of FR) did not tend to internalize folic-AIEgen nanoparticles after 24-hour incubation (top). Scale bars = 50 μm. The experiment was repeated at least three times.

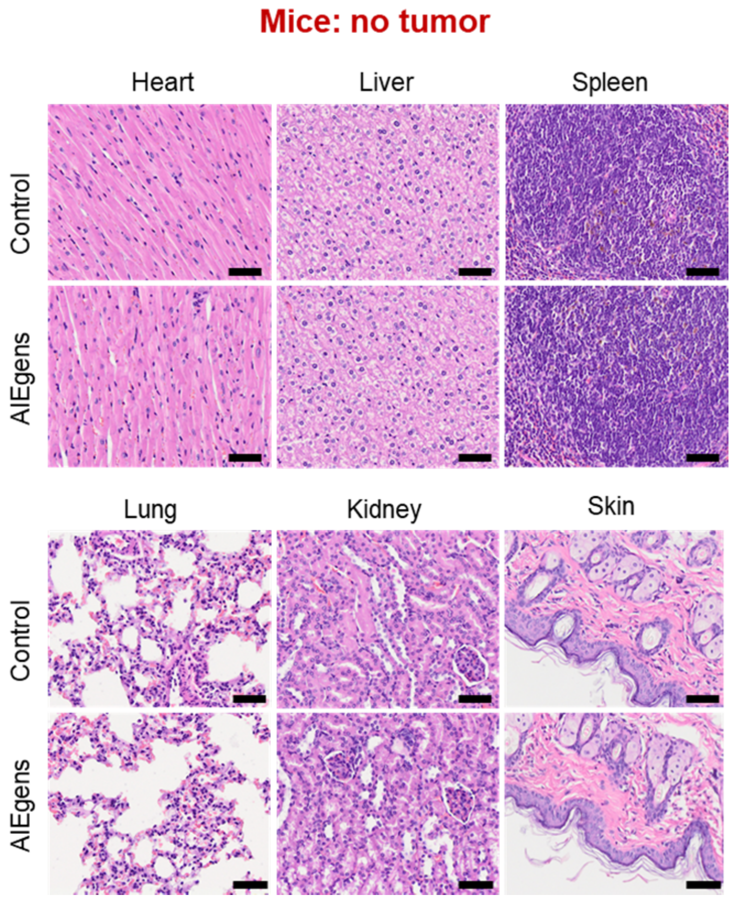


**Supplementary Figure 4. Histological images of the major organs from the mice at 30 days after surgical operation.** Representative H&E staining images of heart, liver, spleen, lung, kidney, and skin. Scale bar = 50 μm. The experiment was repeated at least three times.

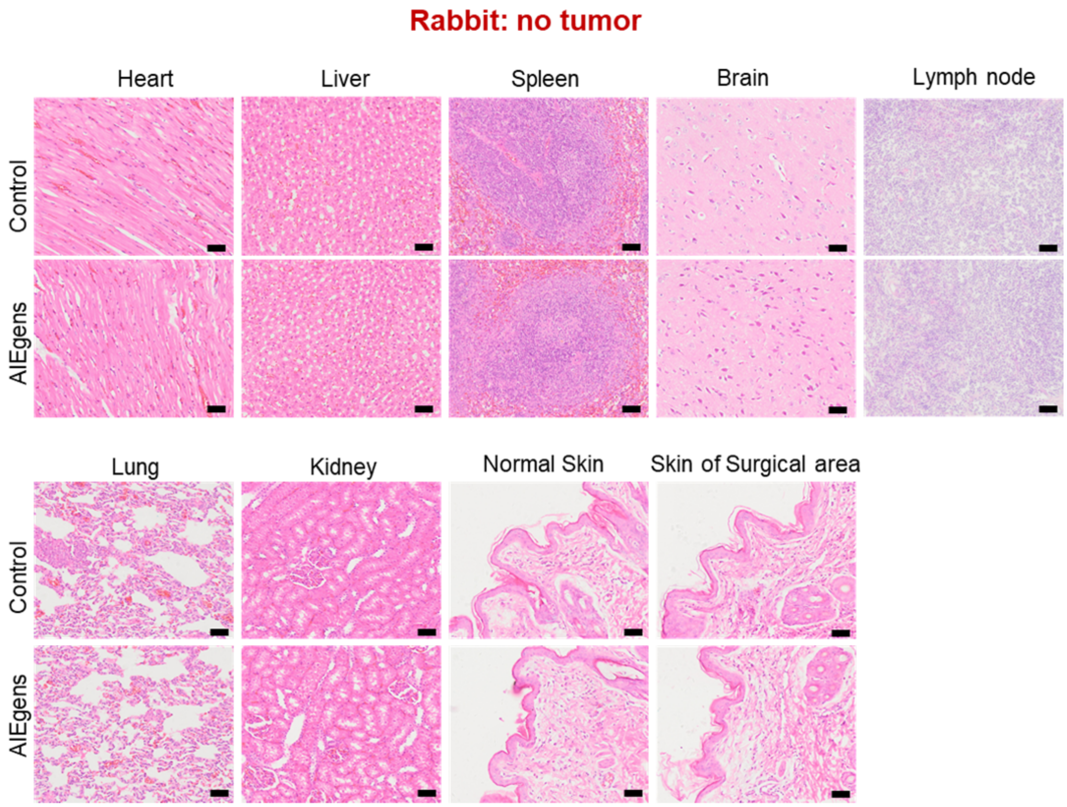


**Supplementary Figure 5. Histological images of the major organs from the rabbits at 30 days after surgical operation.** Representative H&E staining images of heart, liver, spleen, lung, kidney, normal skin, and skin of surgical area. Scale bar = 50 μm. The experiment was repeated at least three times.

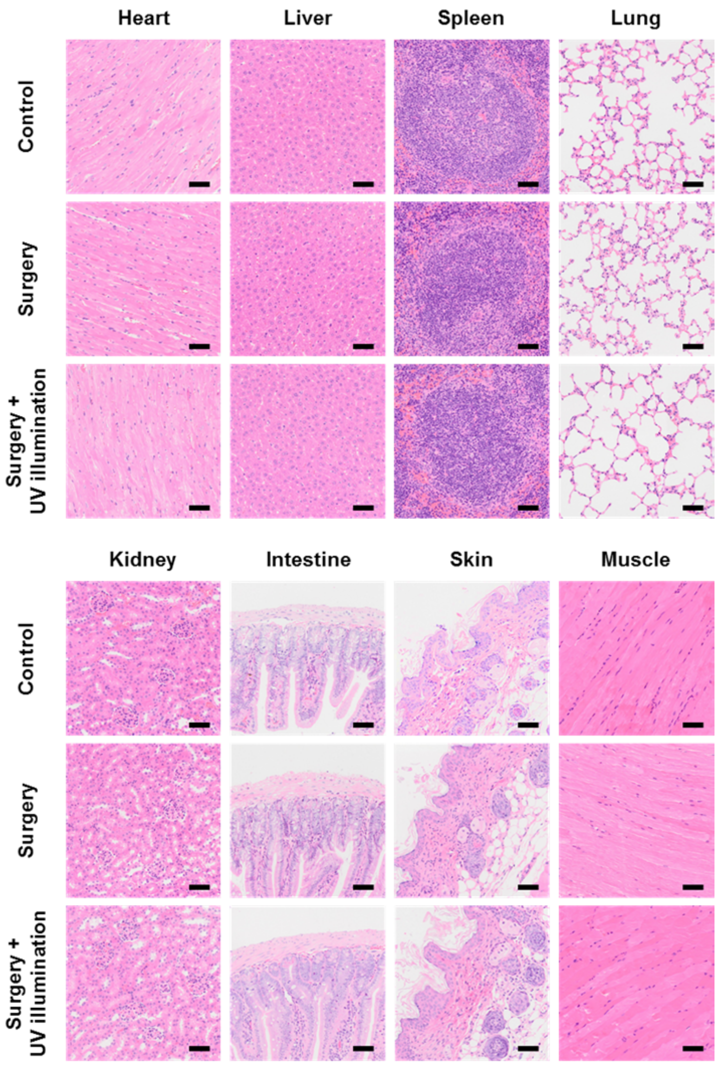


**Supplementary Figure 6. Histological images of the major organs from the mice at 30 days after different treatments.** Representative H&E staining images of heart, liver, spleen, lung, kidney, intestine, skin and muscle (n = 6). Scale bar = 50 μm. The experiment was repeated at least three times.

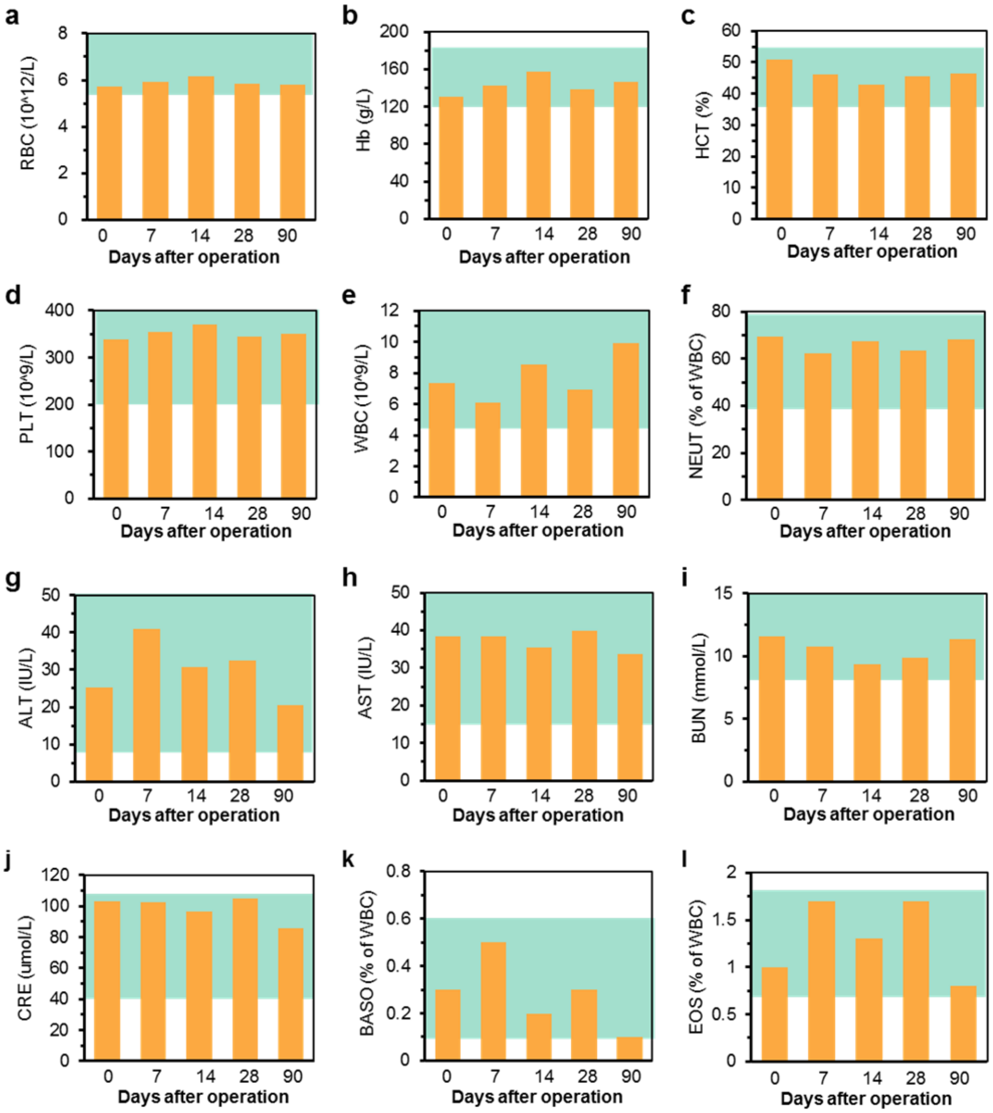


**Supplementary Figure 7. Blood test results (a-l) for the rhesus macaques before and after the surgical operation.** There were no abnormalities in liver and kidney function, complete blood count and immune system over the 90-day post-treatment. Abbreviations: haemoglobin, Hb; red blood cell count, RBC; haematocrit, Hct; platelet count, PLT; white blood cell count, WBC; neutrophil granulocyte, NEUT; lymphocyte, LY; monocyte, MONO; eosinophil granulocyte, EOS; basophil granulocyte, BASO; alanine transaminase, ALT; aspartate transaminase, AST; blood urea nitrogen, BUN; creatinine, CRE. The regions rendered in GREEN represent the normal range. Before the operation, the animal is subject to blood testing as the normal control.

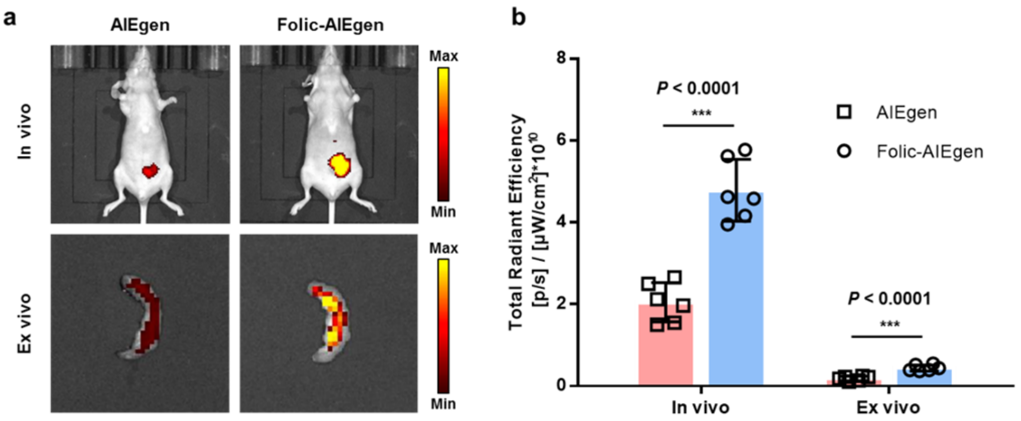


**Supplementary Figure 8. Specific tumor targeting ability of folic-AIEgen in the intraperitoneal SKOV3 xenograft mouse model.** (a) In vivo fluorescence imaging of mice (top) 24 hours after intraperitoneal injection of AIEgen or folic-AIEgen (100 μL, 40 μg/mL) and Ex vivo fluorescence imaging of the intestine tissues with tumors collected from mice (bottom) (n = 6). (b) Quantitative analysis of the fluorescence signals of the in vivo and ex vivo images. Data are means ± SD, n = 6, Student's two-tailed t test, ***$P$ < 0.001.

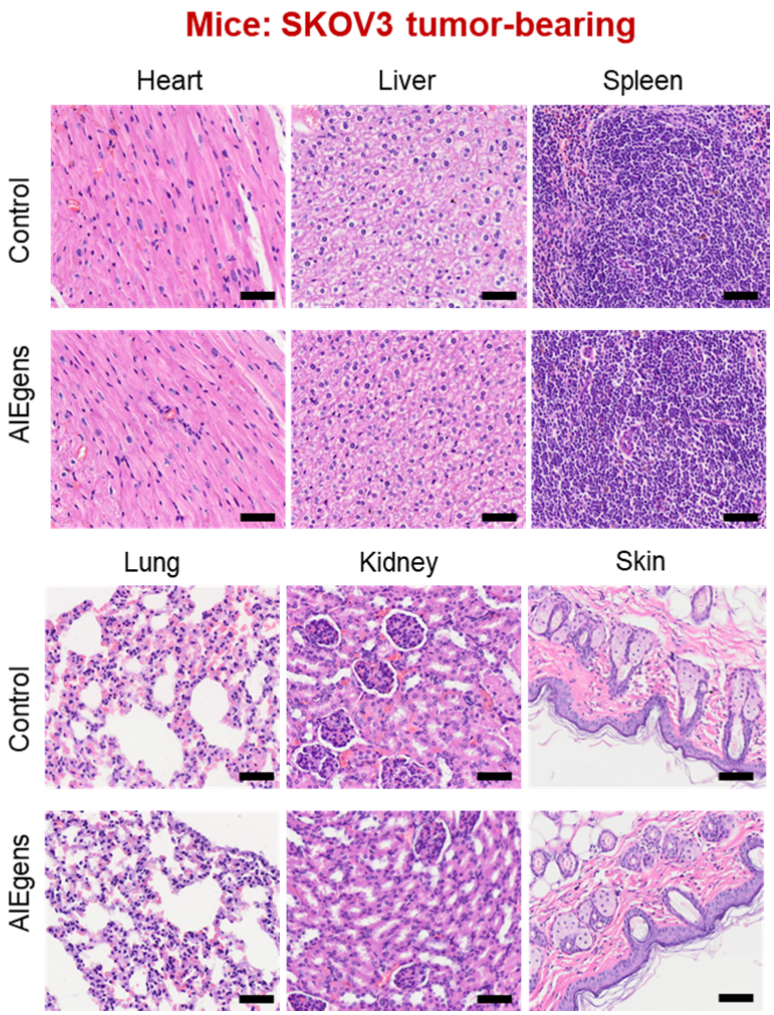


**Supplementary Figure 9. Histological images of the major organs from the tumor-bearing mice at 30 days after surgical operation.** Representative H&E staining images of heart, liver, spleen, lung, kidney, and skin. Scale bar = 50 μm. The experiment was repeated at least three times.

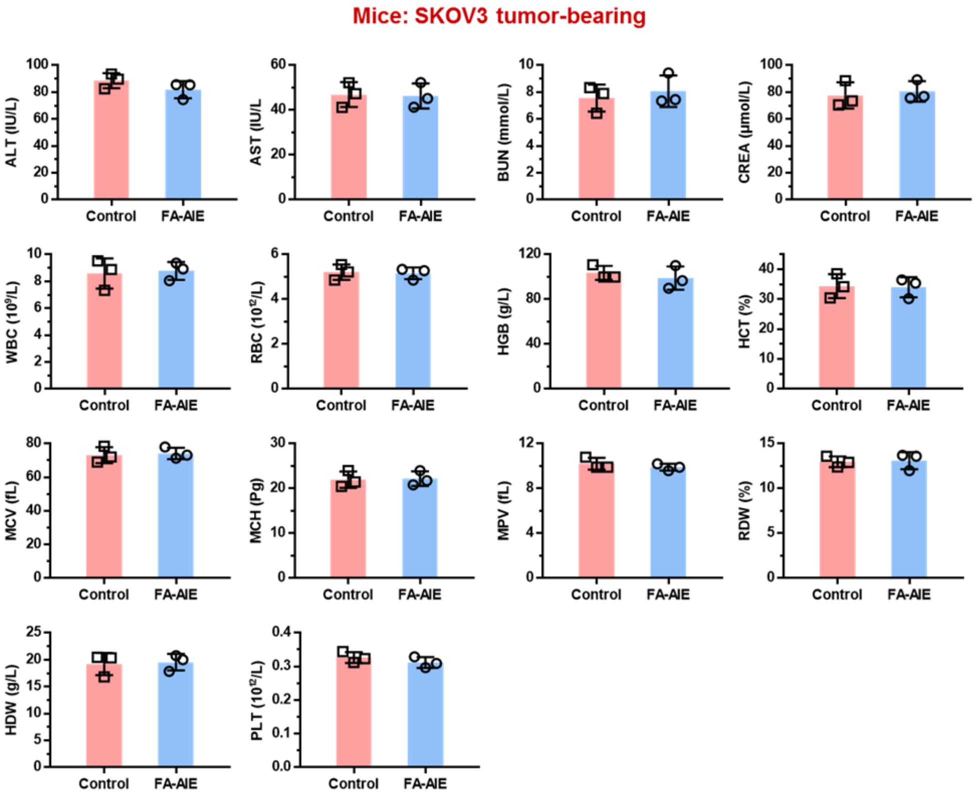


**Supplementary Figure 10. Blood test results for the SKOV3 tumor-bearing mice after intraperitoneal injection of folic-AIEgen.** There were no abnormalities in liver and kidney function, complete blood count and immune system over the 90-day post-treatment. Abbreviations: folic-AIEgen, FA-AIE, haemoglobin, Hb; red blood cell count, RBC; haematocrit, Hct; platelet count, PLT; white blood cell count, WBC; neutrophil granulocyte, NEUT; lymphocyte, LY; monocyte, MONO; eosinophil granulocyte, EOS; basophil granulocyte, BASO; alanine transaminase, ALT; aspartate transaminase, AST; blood urea nitrogen, BUN; creatinine, CRE. The regions rendered in GREEN represent the normal range. Before the operation, the animal is subject to blood testing as the normal control. Data are means ± SD, n = 3.

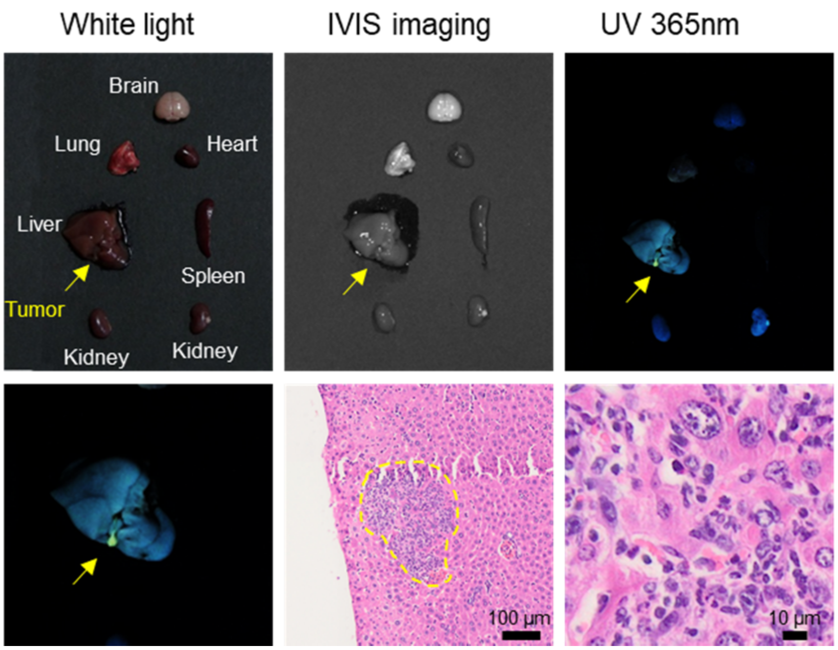


**Supplementary Figure 11. Targeted imaging of AIEgens in metastatic liver tumor.** Ex vivo images of major organs in Hela metastatic tumor-bearing mice under white light, IVIS imaging system, and UV light (top). Ex vivo image of the liver with tumor under UV light and representative H&E staining images (bottom). Yellow arrow and yellow circle: metastatic tumor. The experiment was repeated at least three times.

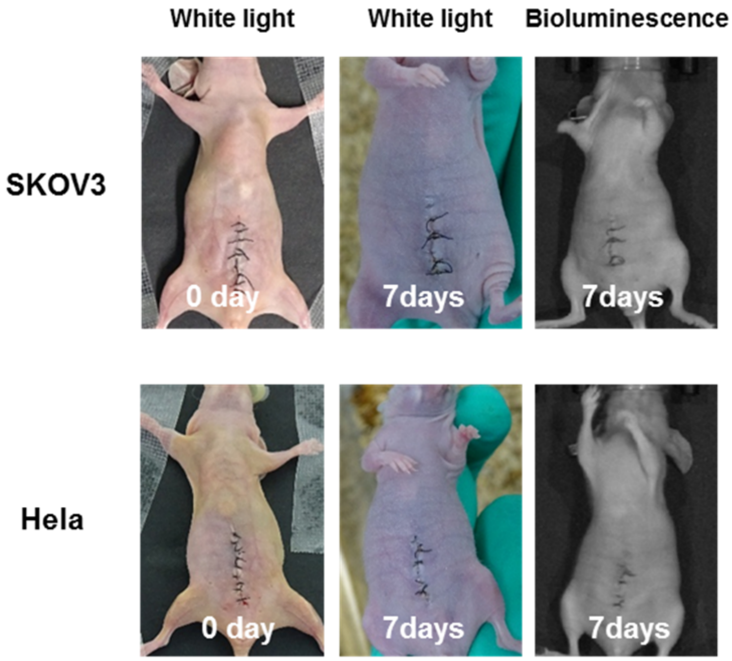


**Supplementary Figure 12. White light and bioluminescence images of intraperitoneal SKOV3 and Hela tumors bearing mice after surgery.** 7 days after surgery, there were no signs of recurrence observed via bioluminescence imaging.

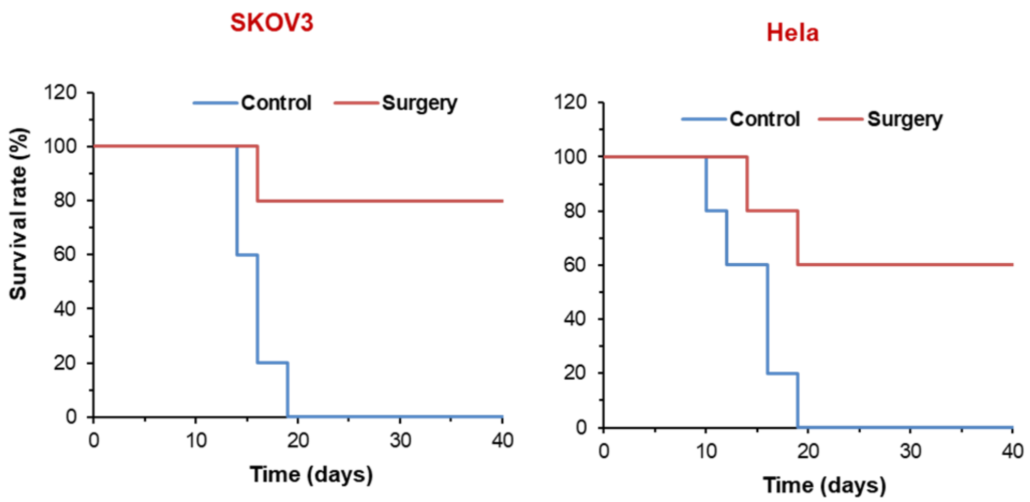


**Supplementary Figure 13. Survival rate of intraperitoneal SKOV3 and Hela tumors bearing mice with or without surgery.**

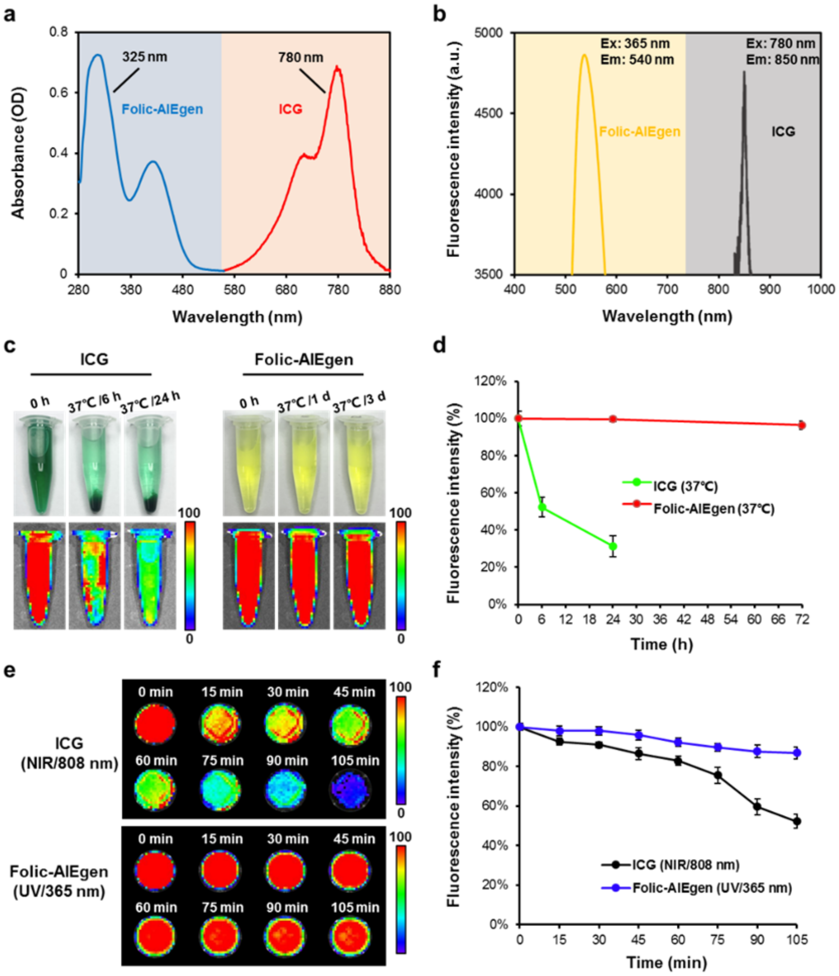


**Supplementary Figure S14. Comparison of fluorescence characteristics and stability between ICG and folic-AIEgen.** (a) UV-vis spectra and (b) fluorescence spectra of ICG and folic-AIEgen. (c) Time-dependent photographs (top) and fluorescence imaging (bottom) of ICG (250 μg/mL) and folic-AIEgen (40 μg/mL) in PBS at 37 °C. (d) Quantitative analysis of the fluorescence signals of ICG and folic-AIEgen in fluorescence imaging. Data are means ± SD, n = 3. (e) Time-dependent fluorescence imaging of ICG (NIR, 808 nm, 250 μg/mL) and folic-AIEgen (365 nm, 40 μg/mL) subjected to continuous laser irradiation. (f) Quantitative analysis of the fluorescence signals of ICG and folic-AIEgen in fluorescence imaging. Data are means ± SD, n = 3.

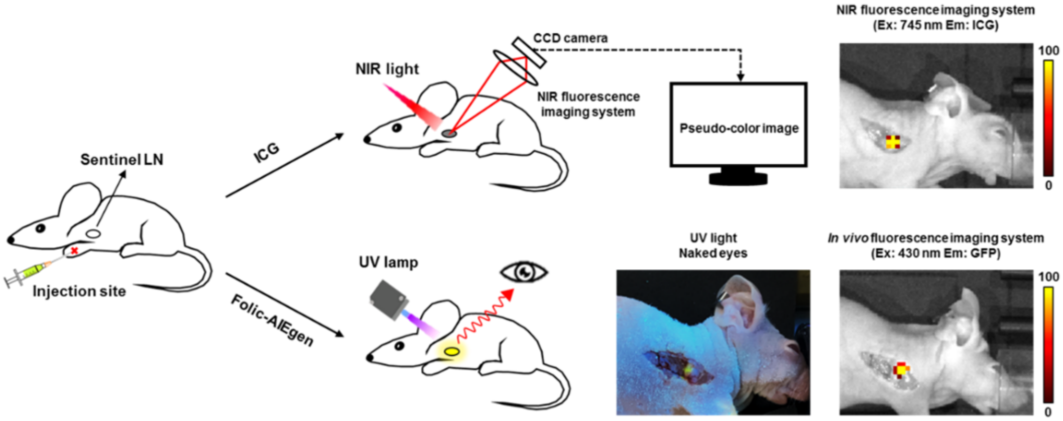


**Supplementary Figure S15. Schematic illustration of ICG and folic-AIEgen guided SLN dissection in nude mice.** NIR fluorescence imaging of ICG (250 μg/mL, 25 μL) administered mice with skin removal was imaged by NIR fluorescence imaging system. Photographs and fluorescence imaging of folic-AIEgen (40 μg/mL, 25 μL) administered mice with skin removal were recorded under UV light and IVIS imaging system, respectively.

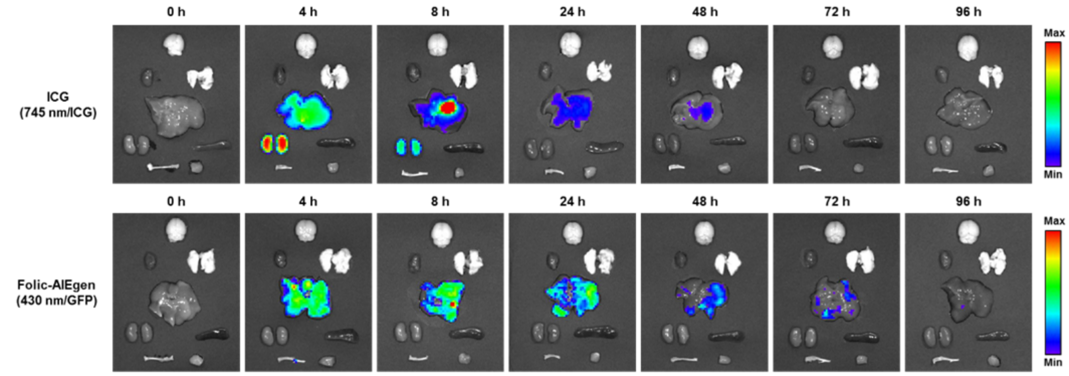


**Supplementary Figure 16. Biodistribution of ICG and folic-AIEgen in nude mice after intravenous administration.** Ex vivo fluorescence imaging of major tissues (brain, heart, lung, liver, kidney, spleen, bone and muscle) of mice (n = 6) collected at different time points after intravenous injection with ICG (250 μg /ml, 100 μL) or folic-AIEgen (40 μg/ml, 100 μL). Images in different groups were required under the corresponding instrumental conditions (Ex: 745 nm/Em: ICG for ICG group, Ex: 430 nm/Em: GFP for folic-AIEgen group).
