# Supplementary material for: Aggregation-induced emission luminogens for image-guided surgery in non-human primates
Source: Nat Commun. 2021 Nov 10;12:6485. doi: 10.1038/s41467-021-26417-2 (PMC9632329; doi:10.1038/s41467-021-26417-2)
Supplement: Supplementary file 3 — Description of Additional Supplementary Files [file 41467_2021_26417_MOESM3_ESM.docx]

File Name: Supplementary Video 1

Description: Folic-AIEgen based image-guided operation for SLN detection and dissection in rabbit.

File Name: Supplementary Video 2

Description: Folic-AIEgen based image-guided operation for SLN detection and dissection in Rhesus macaque’s breast SLNs.

File Name: Supplementary Video 3

Description: Image-guided biopsy for lymph node metastasis of breast cancer in 4T1 breast cancer bearing mice via folic-AIEgen.
